# Assessing the Relationship Between COVID-19 and Dental Decay Risk in Youth: A Retrospective Analysis

**DOI:** 10.7759/cureus.60142

**Published:** 2024-05-12

**Authors:** Pallavi K Chakraborty, Arpan Biswas, Mridul Sharma, Ankur Jyoti Bharali, Nirav Parmar, Sheerja Hemal Shah

**Affiliations:** 1 Oral and Maxillofacial Surgery, Annapurna Health Point Hospital, Durgapur, IND; 2 Public Health Dentistry, Vyas Dental College and Hospital, Jodhpur, IND; 3 Faculty of Dentistry, McGill University, Montreal, CAN; 4 Periodontics and Oral Implantology, Vyas Dental College and Hospital, Jodhpur, IND; 5 Conservative Dentistry and Endodontics, Faculty of Dental Science, Dharmsinh Desai University, Nadiad, IND; 6 Dentistry, AMC (Ahmedabad Municipal Corporation) Dental College and Hospital, Ahmedabad, IND

**Keywords:** dmfs, dmft, decayed missing and filled teeth (dmft), young adults, children, adolescents, dental caries, covid-19

## Abstract

Background: The coronavirus disease 2019 (COVID-19) pandemic has significantly impacted public health globally, with particular attention on the effects on children, adolescents, and young adults. This study aimed to investigate the potential relationship between COVID-19 and caries risk in this age group.

Methods: A retrospective chart review was conducted on 120 patients between the ages of six and 25 years who received dental treatment at a university-affiliated dental clinic between January 2020 and December 2021. Demographic and medical data were collected, and dental examinations were performed to record the number of decayed, missing, and filled teeth (DMFT) and decayed, missing, and filled surfaces (DMFS). Data were analyzed using descriptive statistics, chi-square test or Fisher's exact test, student's t-test or Mann-Whitney U test, and multivariate regression analysis.

Results: Of the 120 patients, 40 were COVID-19-positive, and 32 of those patients were at high caries risk. Of the 80 patients who were COVID-19 negative, 48 were at high caries risk. There was a statistically significant association between COVID-19 exposure and caries risk. Participants who tested positive for COVID-19 had 1.8 times higher odds of dental caries than those who tested negative. However, no significant association was found between caries risk and age, gender, or previous dental history.

Conclusion: The findings suggest that COVID-19 may be a risk factor for caries in children, adolescents, and young adults. Dental professionals should consider COVID-19 exposure as a potential risk factor when assessing caries risk in this age group. Further research is needed to better understand the mechanism underlying this association.

## Introduction

The World Health Organization (WHO) reports that over 760 million cases of coronavirus disease 2019 (COVID-19) have been confirmed worldwide, resulting in an estimated 6.9 million fatalities [1]. While the disease has impacted people of all ages globally, its prevalence and severity vary with age. According to WHO data, adults are more commonly affected by COVID-19 compared to children. Despite this trend, concern regarding the prevalence and potential consequences of COVID-19 in pediatric patients has escalated in recent years. COVID-19 is a highly contagious respiratory illness caused by the novel coronavirus severe acute respiratory syndrome coronavirus 2 (SARS-CoV-2) [1]. While the disease primarily affects the respiratory system, growing evidence suggests that COVID-19 may also have implications for oral health [2]. Poor oral health, including dental caries, is a significant public health issue among children, adolescents, and young adults [3].

Several studies have reported a potential association between COVID-19 and oral health. For example, a systematic review and meta-analysis found that COVID-19 patients had a higher prevalence of oral manifestations, such as taste disturbances and dry mouth, than non-COVID-19 patients [4]. It was also found that COVID-19 patients had higher levels of oral bacteria associated with periodontal disease than healthy controls [5]. However, to our knowledge, no study has specifically investigated the association between COVID-19 and dental caries in children, adolescents, and young adults. Therefore, this study aimed to investigate whether COVID-19 is a risk factor for public health problems in children, adolescents, and young adults.

To address this research gap, we conducted a cross-sectional study on children, adolescents, and young adults. We hypothesized that COVID-19 would be associated with an increased risk of dental caries in these age groups. We collected data on participants' COVID-19 status, oral hygiene habits, dietary habits, and dental caries status. Our findings have implications for public health policies aimed at promoting oral health in children, adolescents, and young adults affected by COVID-19.

## Materials and methods

This was a cross-sectional study conducted to investigate the association between COVID-19 and dental caries in children, adolescents, and young adults. The study protocol was approved by the Institutional Review Board of Annapurna Health Point Hospital (approval number: IEC/AHPH/2022/H-11). The study was conducted in accordance with the principles of the Declaration of Helsinki. Informed consent was obtained from each participant or their legal guardian before participation in the study.

Inclusion and exclusion criteria

Children, adolescents, and young adults aged 6-25 years who received dental treatment at a university-affiliated dental clinic, Annapurna Health Point Hospital, Durgapur, India, between January 2020 and December 2021, who are from areas with a high prevalence of COVID-19, and who provided informed consent, or whose legal guardians provided consent on their behalf, were included in the study. Individuals from areas with low or moderate COVID-19 prevalence were excluded from the study.

Sample size

The sample size was determined based on the prevalence of COVID-19 in the study area and the anticipated response rate. The sample size was calculated using statistical power analysis to ensure adequate power for the study using a formula

n=Z^2^×p×(1−p)​/d2

Where, n = required sample size, Z-score corresponding to the desired level of confidence (e.g., 1.96 for a 95% confidence level), p = estimated prevalence or proportion of the population with the characteristic of interest, d = margin of error (also known as precision).

Data collection

Demographic data and medical history, including age, sex, COVID-19 symptoms, medical conditions, medication use, oral hygiene habits, and dietary habits, were collected from each participant by a trained research assistant. The research assistant also obtained information on the COVID-19 test results from medical records.

Dental examinations were performed under artificial light by two calibrated examiners, using dental mirrors and probes. Prior to the start of the study, the examiners underwent training and calibration exercises to ensure the consistency and reliability of the measurements. The examiners recorded the number of decayed, missing, and filled teeth (DMFT) and decayed, missing, and filled surfaces (DMFS) for each participant according to WHO criteria [6].

All examinations were conducted while adhering to strict infection control measures to minimize the risk of COVID-19 transmission. The examiner wore personal protective equipment, including masks, gloves, and gowns, and sterilized all equipment between participants.

Data analysis

Descriptive statistics were used to summarize the data. The chi-square test or Fisher's exact test was used to compare categorical variables between COVID-19-infected and non-infected participants. The Mann-Whitney U test was used to compare continuous variables between the two groups. Multivariate regression analysis was used to assess the association between COVID-19 and dental caries while controlling for confounding factors.

Data management

All data were stored in a secure database with restricted access to authorized personnel. Data were anonymized and de-identified to ensure confidentiality.

## Results

A total of 120 children, adolescents, and young adults aged 6-25 years were included in the study. Table 1 presents the characteristics of the study participants, including their age, gender, oral hygiene habits, and dietary habits. Of the 120 participants, 40 (33.3%) participants tested positive for COVID-19 infection and 80 (66.7%) participants tested negative. There was a relatively even split between males and females in both the COVID-19-positive and COVID-19-negative groups. The majority of participants reported brushing their teeth at least twice a day, and around half reported consuming sugary snacks and drinks at least once a day. 

**Table 1:** Characteristics of study participants

Characteristics	COVID-19 Positive (n=40)	COVID-19 Negative (n-80)
Age (years), mean ± SD	15.5 ± 4.2	14.8 ± 3.9
Gender, n (%)	22 (55.0%)	42 (52.5%)
Male	22 (55.0%)	42 (52.5%)
Female	18 (45.0%)	38 (47.5%)
Oral Hygiene Habits, n (%)		
Brushing ≥2x/day	31 (77.5%)	62 (77.5%)
Flossing ≥1x/day	10 (25.0%)	28 (35.0%)
Dietary Habits, n (%)		
Sugary snacks ≥1x/day	28 (70.0%)	44 (55.0%)
Sugary drinks ≥1x/day	18 (45.0%)	31 (38.8%)

Table 2 shows the dental caries status of study participants, as measured by the DMFT and DMFS scores. The DMFT score represents the number of decayed, missing, and filled permanent teeth, while the DMFS score represents the number of decayed, missing, and filled tooth surfaces. Participants who tested positive for COVID-19 had higher mean DMFT and DMFS scores compared to those who tested negative, indicating a higher prevalence of dental caries in the COVID-19-positive group.

**Table 2:** Dental caries status among study participants DMFT: decayed, missing, and filled teeth; DMFS: decayed, missing, and filled surfaces

Dental Caries Status	COVID-19 Positive (n=40)	COVID-19 Negative (n=80)
DMFT Score, mean ± SD	4.6 ± 2.1	3.2 ± 1.6
DMFS Score (mean ± SD)	6.5 ± 3.1	4.3 ± 2.4
Dental Caries, n (%)		
Yes	32 (80.0%)	48 (60.0%)
No	8 (20.0%)	32 (40.0%)

Table 3 presents the association between COVID-19 infection and dental caries. The odds ratio (OR) represents the likelihood of having dental caries in the COVID-19-positive group compared to the COVID-19-negative group. Participants who tested positive for COVID-19 had 1.8 times higher odds of dental caries compared to those who tested negative. The p-value indicates that this association was statistically significant (p < 0.05).

**Table 3:** Association between COVID-19 infection and dental caries

COVID-19 Infection	Dental Caries, n (%)	Odds Ratio (95% CI)	p-Value
Positive	32 (80.0%)	1.8 (1.1-3.0)	0.02
Negative	48 (60.0%)	Reference	-

Table 4 shows the association between COVID-19 severity and dental caries. Participants who experienced severe COVID-19 symptoms had higher mean DMFT and DMFS scores compared to those who experienced mild to moderate symptoms, indicating a higher prevalence of dental caries in the severe COVID-19 group.

**Table 4:** Association between COVID-19 severity and dental caries DMFT: decayed, missing, and filled teeth; DMFS: decayed, missing, and filled surfaces; COVID-19: coronavirus disease 2019

COVID-19 Severity	DMFT Score, mean ± SD	DMFS Score, mean ± SD
Mild to Moderate (n=25)	4.2 ± 2.0	5.9 ± 2.9
Severe (n=15)	5.8 ± 2.7	8.1 ± 4.2

## Discussion

COVID-19 is a highly contagious respiratory illness caused by the novel coronavirus SARS-CoV-2 [7]. While the disease primarily affects the respiratory system, growing evidence suggests that COVID-19 may also have implications for oral health [8,9]. Poor oral health, including dental caries, is a significant public health issue among children, adolescents, and young adults [10].

This study aimed to investigate whether COVID-19 is a risk factor for public health in children, adolescents, and young adults. Our findings suggest that there is a positive association between COVID-19 and dental caries. Participants who tested positive for COVID-19 had higher mean DMFT and DMFS scores than those who tested negative. Additionally, participants who experienced severe COVID-19 symptoms had higher mean DMFT and DMFS scores than those who experienced mild to moderate symptoms.

Our results are consistent with those of previous studies that have reported an association between COVID-19 and oral health. For instance, a study by Muthyam et al. found that COVID-19 patients had a significantly higher prevalence of dental caries than healthy controls [11]. Similarly, Cagna et al. reported that patients with taste disturbances due to COVID-19 had a higher prevalence of dental caries [12]. Another study by Sari et al. reported that COVID-19 patients had a higher prevalence of periodontitis than healthy controls [13].

One possible explanation is that COVID-19 may lead to changes in oral microbiota, which could contribute to the development of dental caries [14]. The oral microbiota plays an essential role in maintaining oral health and changes in the microbiota can lead to the development of dental caries [15]. COVID-19 patients may also experience dry mouth or changes in taste, which could affect their dietary habits and increase their risk of dental caries [16]. Additionally, COVID-19 patients may be taking medications that affect their oral health, such as antibiotics, antivirals, and corticosteroids, which can alter the oral microbiota and increase the risk of dental caries [17].

Our findings have important implications for public health policies aimed at promoting oral health among children, adolescents, and young adults affected by COVID-19. Dental health professionals should be aware of the potential association between COVID-19 and dental caries and should encourage patients to maintain good oral hygiene and dietary habits. Additionally, public health campaigns should emphasize the importance of oral health during the COVID-19 outbreaks, particularly for individuals who test positive for COVID-19.

The limitations of this study include its small sample size and cross-sectional design, which precludes us from drawing causal conclusions. The dental history's crucial factor, previous DMFS, carries risk assessment was not mentioned. The bias present lies in the observation that COVID-positive patients tend to consume a higher quantity of sugary snacks since sugar is a significant contributing factor to dental cavities. Future studies should investigate the longitudinal association between COVID-19 and dental caries, and explore the potential mechanisms underlying this association.

## Conclusions

This study found a statistically significant association between COVID-19 exposure and caries risk in children, adolescents, and young adults. These results suggest that COVID-19 may be a risk factor for caries in this age group, and that dental professionals should consider COVID-19 exposure as a potential risk factor when assessing caries risk in their patients. However, no significant association was found between caries risk and age, sex, or dental history. Further research is needed to better understand the underlying mechanism of this association and explore potential preventive measures to mitigate the impact of COVID-19 on oral health.
